# Standardising costs or standardising care? Qualitative evaluation of the implementation and impact of a hospital funding reform in Ontario, Canada

**DOI:** 10.1186/s12961-018-0353-6

**Published:** 2018-08-03

**Authors:** Karen S. Palmer, Adalsteinn D. Brown, Jenna M. Evans, Husayn Marani, Kirstie K. Russell, Danielle Martin, Noah M. Ivers

**Affiliations:** 1 Women’s College Research Institute, Women’s College Hospital, 76 Grenville St, Toronto, ON M5G 1N8 Canada; 2 Faculty of Health Sciences, Simon Fraser University, Burnaby, BC Canada; 3 Institute of Health Policy, Management and Evaluation, Dalla Lana School of Public Health, University of Toronto, Toronto, ON Canada; 4 Li Ka Shing Knowledge Institute, St. Michael’s Hospital, Toronto, ON Canada; 5 Enhanced Program Evaluation Unit, Cancer Care Ontario, Toronto, ON Canada; 6 Institute for Health System Solutions and Virtual Care, Women’s College Hospital, Toronto, ON Canada; 7 Department of Family and Community Medicine, Women’s College Hospital and University of Toronto, Toronto, ON Canada

**Keywords:** Organisational change, Organisational implementation strategies, Hospital funding reform, Healthcare policy

## Abstract

**Background:**

Since 2011, the Government of Ontario, Canada, has phased in hospital funding reforms hoping to encourage standardised, evidence-based clinical care processes to both improve patient outcomes and reduce system costs. One aspect of the reform – quality-based procedures (QBPs) – replaced some of each hospital’s global budget with a pre-set price per episode of care for patients with specific diagnoses or procedures. The QBP initiative included publication and dissemination of a handbook for each of these diagnoses or procedures, developed by an expert technical group. Each handbook was intended to guide hospitals in reducing inappropriate variation in patient care and cost by specifying an evidence-based episode of care pathway. We explored whether, how and why hospitals implemented these episode of care pathways in response to this initiative.

**Methods:**

We interviewed key informants at three levels in the healthcare system, namely individuals who conceived and designed the QBP policy, individuals and organisations supporting QBP adoption, and leaders in five case-study hospitals responsible for QBP implementation. Analysis involved an inductive approach, incorporating framework analysis to generate descriptive and explanatory themes from data.

**Results:**

The 46 key informants described variable implementation of best practice episode of care pathways across QBPs and across hospitals. Handbooks outlining evidence-based clinical pathways did not address specific barriers to change for different QBPs nor differences in hospitals’ capacity to manage change. Hospitals sometimes found it easier to focus on containing and standardising costs of care than on implementing standardised care processes that adhered to best clinical practices.

**Conclusion:**

Implementation of QBPs in Ontario’s hospitals depended on the interplay between three factors, namely complexity of changes required, internal capacity for organisational change, and availability and appropriateness of targeted external facilitators and supports to manage change. Variation in these factors across QBPs and hospitals suggests the need for more tailored and flexible implementation supports designed to fit all elements of the policy, rather than one-size-fits-all handbooks alone. Without such supports, hospitals may enact quick fixes aimed mainly at preserving budgets, rather than pursue evidence- and value-based changes in care management. Overestimating hospitals’ change management capacity increases the risk of implementation failure.

**Electronic supplementary material:**

The online version of this article (10.1186/s12961-018-0353-6) contains supplementary material, which is available to authorized users.

## Background

Health systems around the world are responding to pressures to achieve the Triple Aim [1] by testing new approaches to funding healthcare services. The underlying assumption is that funding models can be used as a lever to encourage the implementation of better care processes [2].

In 2011, the Government of Ontario, Canada, began a multi-year implementation of hospital funding reforms. These reforms included replacing some of each hospital’s global budget with procedure- or diagnosis-specific funding based on a pre-set price per episode of care, a variant of activity-based funding [3]. This aspect of the overall funding reform was named ‘quality-based procedures’ (QBPs). A small number of diagnoses and procedures chosen for this type of care-episode funding were phased in over time (Additional file 1). Specific diagnoses and procedures were selected for the QBP initiative based on perceived potential to reduce variation through implementation of best practice recommendations and indicators to monitor for ongoing quality improvement. Designers of QBPs have espoused a variety of goals, including improving access, efficiency, transparency and clinician engagement, and reducing variation in treatment costs, treatment processes and/or clinical outcomes. However, perceptions of the primary goal of QBPs seem to vary across health system stakeholders [4].

To support the implementation of QBPs, the provincial government, in collaboration with Health Quality Ontario (provincial advisor on quality of healthcare) and/or relevant disease-oriented agencies, developed and published a series of handbooks – one for each QBP – outlining best practice episode of care pathways for managing patients with particular diagnoses or undergoing particular procedures. These handbooks and the episode of care pathways contained therein –developed in partnership with technical expert advisory panels including leading clinicians, scientists and patients – serve as a compendium of evidence and clinical consensus for each QBP. Each QBP clinical handbook defines a target patient cohort for a particular diagnosis or procedure and recommends an evidence-based episode of care pathway for that cohort. This includes the critical decision points and phases of treatment for implementation in a particular care setting (e.g. pre-hospital, acute care and post-acute care). Each handbook also suggests quality indicators to monitor the impact of changes made to implement the episode of care pathway for the targeted cohort. During the period covered by our study (2012–2017), 15 handbooks were produced and published (Additional file 1) for funded QBPs (additional handbooks were subsequently produced for QBPs contemplated but not yet separately funded) [5]. The methodology and evidence underlying the episode of care pathways is extensively described in each handbook. For example, the QBP clinical handbook for hip fracture contains the evidence, rationale and clinical consensus for the care of hip fracture patients seen in hospitals [6], including evidence-based health technology assessment, development of cohort and patient group definitions, synthesis of credible national and international guidelines with attention to the strength of evidence supporting each, rapid evidence review where an evidentiary gap was identified, descriptive and multivariate analysis of empirical data, development of measurement frameworks to manage and track actual performance against the recommended practices outlined in the episodes of care, and engagement with clinical experts to guide and inform decisions. Each of the handbooks for the other QBPs repeated this highly sophisticated methodology and analysis. These 15 handbooks, along with an implementation toolkit [7] describing generic steps to put the handbooks into practice, constituted the primary adoption supports provided to hospitals during the period of our study.

Previous research [4] has evaluated the degree to which the QBP policy demonstrated implementation fidelity [8]. To date, there has been no formative evaluation of the implementation process itself or of the extent to which adoption supports used to encourage uptake of QBPs in hospitals were successful. In the absence of such an evaluation, we cannot know whether the adoption supports helped or hindered implementation, or how to spread and scale-up desired transformational changes beyond the jurisdiction where QBPs were first implemented [9, 10]. Our research, therefore, aimed to address this knowledge gap by exploring the processes through which QBPs were implemented in hospitals and how these processes could be supported and enhanced in future.

## Methods

Using an embedded case study method [11], we undertook in-depth, one-on-one, semi-structured telephone interviews with key informants at each of three levels of the healthcare system, namely policy designers (Level 1), comprising individuals who conceived of and were involved in the design of the QBP policy; adoption supporters (Level 2), comprising individuals in external organisations supporting QBP adoption; and hospital implementers (Level 3), comprising individuals in hospitals who are responsible for QBP implementation. All interviews were voluntary and confidential. The Women’s College Research Institute Research Ethics Board approved the study (REB# 2016–0016-E). Methods have been previously described in detail elsewhere [4].

### Sampling and recruitment

Using purposive sampling, we sought information-rich cases while ensuring broad representation across three levels of stakeholders. To identify policy designers (Level 1) and adoption supporters (Level 2), we reviewed public documents about the hospital funding reform and consulted with research team members familiar with the reform to generate a list of potential key informants. One team member (AB) directly emailed these individuals to request their participation and to facilitate communication between them and the interviewer (KP).

To identify hospital implementers (Level 3), we selected hospitals through a multiple-stage, stratified, purposeful, sampling approach, aided by Qualtrics^®^ online survey software. First, we surveyed executives in 14 Local Health Integration Networks (responsible for planning, integrating and funding local healthcare), the Ontario Hospital Association and Cancer Care Ontario (a provincial agency and primary adviser on cancer services, with a province-wide initiative to monitor and improve wait times for key healthcare services [12, 13]). We asked survey respondents to confidentially identify and justify three higher performing hospitals with regard to QBPs and three lower performing hospitals with the most room for improvement, from among the 71 hospitals known to be implementing QBPs. Within each of the (perceived) higher/lower performing categories, we then stratified according to academic and community hospitals, and further stratified community hospitals by size (large vs. small) and geographic location (rural vs. urban). We ultimately selected five representative hospitals, including three academic/teaching hospitals (two higher performing; one lower) and two community hospitals (one higher performing; one lower).

One research team member (AB) then contacted each representative hospital’s Chief Executive Officer (CEO) to seek permission to include their facility in our research. If they agreed, we then asked the CEO to connect our team with leaders or their delegates. We sought interviews within each of the following four groups: (1) chief executive (e.g. CEO); (2) financial/decision support (e.g. Chief Financial Officer); (3) clinical (e.g. VP/Executive VP Clinical Affairs); and (4) medical (e.g. VP Medical Affairs). If initial informants nominated other participants, we pursued snowball sampling to achieve saturation.

### Data collection and management

One research team member (KP) conducted all interviews by telephone, following a semi-structured interview guide. We pre-tested the interview guide and continued to refine it iteratively based on regular discussion with the research team regarding emerging findings of interest. We sought further information at all three levels and within each case-study hospital until saturation was reached. Interviews were audio recorded, professionally transcribed and de-identified. The interviewer made summary case notes immediately after each interview.

### Analysis approach

We used thematic analysis [14] involving an inductive approach, incorporating Framework analysis [15] to generate descriptive and explanatory themes as they emerged from the data. The analysis process included familiarisation with the data by immersion in it, identification of a thematic framework to guide coding, iterative refinement of the codebook, indexing and charting of the data, and mapping/interpretation [16, 17]. Themes were generated from the data through open (unrestricted) coding to create a template of codes. Two research team members (KP, JE) independently open coded two test transcripts to generate themes, then collaboratively reconciled and revised their coding. Then, using this template of codes, two other research team members (HM, KR) independently coded three key informant interview transcripts, comparing and reconciling their coding until they achieved at least 70% agreement on code selection. At that point, we abandoned duplicate coding because of high coherence of coding, but coders regularly consulted with senior members of the research team, including whenever coding questions arose. During weekly team meetings, as new concepts became apparent, we continued to iteratively refine the codebook until no additional codes emerged. We used line-by-line coding and constant comparative methods, assisted by Quirkos^®^ qualitative data analysis software, to facilitate coding, analysis and data management. Each member of the research team independently identified key themes for discussion; upon consensus, data were curated accordingly. Finally, we organised key themes by level of respondent (i.e. levels 1, 2, 3) and by case-study hospital to facilitate comparisons. One research team member (KP) identified an initial set of representative quotations from the transcripts for each key theme. From these quotes, we collaboratively and iteratively sought meaning from the data by building a logical chain of evidence that was conceptually and theoretically coherent [18]. Finally, to ensure validity of the findings, we employed a number of techniques, including researcher reflexivity, peer debriefing, member checking with selected Level 1 and 2 informants, and disconfirming evidence [19, 20].

## Results

We interviewed 46 key informants between May 18 and October 6, 2016, including 12 designers (Level 1), 11 adoption supporters (Level 2), and 22 hospital implementers (Level 3, from 5 case study hospitals). There were no refusals. Interviews ranged from 43 to 107 minutes in length. In the representative quotes presented here, the research team member is designated as ‘R’ and the key informant participants as ‘P’.

### MAIN FINDING 1: Implementation supports provided by the government or other organisations were identical for each QBP and did not address differences in hospitals’ highly variable capacity to manage change

Internal readiness for change (i.e. individual and collective attitudes, beliefs and intentions regarding change [21]) and change management capacity (i.e. structures and resources needed to implement and sustain a change [21]) played a key role in determining the extent to which QBP implementation was successful. Hospitals in which there was a high degree of readiness and change management capacity were better able to use the episode of care pathways in the handbooks to facilitate implementation of standardised care processes. Change management capacity in those hospitals was enabled by pre-existing quality improvement (QI) infrastructure such as standing committees supported by internal data analytics.

“*Some of the hospitals – where we know they’ve invested in developing their team, developing their capacities for quality improvement, their board is already focused on quality improvement, they take their quality improvement process seriously, the quality committee is engaged at the board level, leadership is engaged – in that kind of a culture, it will be easier for them to take on a new priority QBP implementation than in a hospital that hasn’t done that ground work within their organisation already.*”

Source: QBP Designer, Ministry of Health, Level 1 – 006

“*The capacity to even think this through, have the data capacity, I think it’s highly variable. I think the big academic hospitals have been on it. They’ve got mature decision support systems, they’ve got mature case costing systems, they’ve got in-depth clinical leadership, and many of them were on the panels. I think that they are in a very different place than many of the other community hospitals that really have very, very thin decision support capability, and not necessarily a tonne of depth around quality and clinical quality*.”

Source: Senior Executive, QBP Adoption Supporter, Level 2 – 015

“P: *ALC* [Alternative Level of Care] *numbers have gone up in the last couple of weeks so I'm challenging my teams to do something about that.* R: *That seems to be the thing that you're spending most of your attention on. It’s not QBPs so much…* P: *Definitely, no.* R: *Yeah, okay, I understand.* P: *If I can solve that…QBPs is the least of my worries at this time*.”

Source: Senior Executive, Hospital, Level 3 – 026

“*… in terms of the systems of data analytics that are required to really do it well… we don’t have some of the key enablers that will be necessary to really get us to perfection or a lot closer to perfection than we are today*.”

Source: Senior Executive, Hospital, Level 3 – 024

#### Sub-finding 1a: Some QBPs were more difficult to implement than others, requiring unique adoption supports to support change management

The extent to which episode of care pathways were implemented varied not only across hospitals, but also across QBPs. Medically complex, unplanned QBPs were inherently more difficult to implement than planned, procedurally oriented, QBPs. Medically complex QBPs with a high degree of clinical uncertainty required more sophisticated and clinically nuanced adoption supports to initiate the recommended pathway, but these were not available.

“*Then there are two other QBP things that we’re wrestling with. One is heart failure, and the other one is pneumonia. We’ve had a lot of trouble in this hospital, getting people to put patients into those QBPs.* R: *What do you mean by, put patients into those QBPs?* P: *Enrol them into the pathway.* R: *Okay, so follow the pathway?* P: *Yeah, because people come in, and they don’t say, I’ve got heart failure, they come in and say, I’ve got shortness of breath, so we need a shortness of breath pathway. Then blood work is done, an x-ray is done, we figure out, oh, it was actually an infarct that caused the shortness of breath, or it was pneumonia, or it was heart failure, or it was a chest infection that produced a bit of heart failure, so where do you put that patient? When you get away from a very well-described thing like renal failure, a hip replacement, we’ve had a lot of difficulty, and I know other places have had a lot of difficulty with these medical ones*.”

Source: Senior Executive, Hospital, Level 3 – 028

“*When you have the nice, neatly defined episode of care, it’s pretty easy, like hip fractures, knees, and surgical things. They lend themselves very well to QBPs. It works well. The medical ones are more of a challenge, like CHF* [congestive heart failure]*, COPD* [chronic obstructive pulmonary disease]*. The hospital is only a small component of that person’s healthcare journey… the way our system is designed and organised, we have precious little influence or control over probably 90% of their healthcare experience.*”

Source: Senior Executive, Hospital, Level 3 – 022

#### Sub-finding 1b: External QI adoption supports enabled QBP implementation

Pre-existing, external QI and change management supports facilitated implementation of some disease-specific QBPs, but not all. In almost every case, these pre-existing, external QI supports were associated with provincial organisations experienced in supporting change management processes. These included established disease-specific agencies, pre-existing communities of practice, and/or established performance monitoring and evaluation mechanisms. QBPs for which there were no pre-existing, external QI supports were more challenging to implement.

“*I think a key enabler is having some sort of an agency or focus where you know that you’ve already got traction in the system. For example, for our cardiac and vascular QBPs, we already work with those end users and stakeholders. And so, when it came time for us to go out and do some dissemination and some road shows to each of the hospitals, we already had those connections.*”

Source: Senior Executive, QBP Adoption Supporter, Level 2 – 021

“*We know that the first wave of QBPs that were created, which were, of course, closely linked to the original wait times activity, that those are in very advanced stages of adoption. But, subsequent QBPs, subsequent batteries of QBPs, at a minimum we know are implemented in a highly non-standardised manner across the province, and we know that in some parts of the province they’re not implemented at all.”*

Source: Senior Executive QBP Adoption Supporter, Level 2 – 020

“*Having some of those provincial organisations out there assisting hospitals with less internal capacity is a good thing, and I think the OHA* [Ontario Hospital Association] *has also started to do some more analytics support for its organisations, especially small hospitals. So, I think all those are good strategies at the provincial level, to ensure people don’t get left behind.”*

Source: Senior Executive, QBP Adoption Supporter, Level 1 – 014

“*All of the things that the Ontario Stroke Network has been able to do around the stroke QBP are just totally the sorts of things that have contributed to success in terms of adopting clinical best practices. One of the things to remember is that there’s nothing magical about the stroke handbook. Pretty much all the recommendations from that were taken from the existing Heart & Stroke guideline. But, I think the Stroke Network was very adept in terms of capitalising on the QBP introduction, to sort of make this case for clinical practice change, and so they did very well as a result whereas, if you look at COPD, there was much less of that going on. And, I think pneumonia was…a perfect example in that there’s no Ontario pneumonia network. There wasn’t much activity going on in that area, there’s no pneumonia scorecard, any of this stuff brought in feedback. And, so there’s actually probably been very little impact of that pneumonia QBP handbook in terms of clinical practice change.*”

Source: Source: Senior Executive, QBP Adoption Supporter, Level 1 – 014

#### Sub-finding 1c: Adoption supports were lacking to facilitate collaboration among physicians, and between physicians and the broader clinical care teams, in applying best practice episode of care pathways

Regardless of hospitals’ internal capacity for change, and irrespective of whether external QI and change management supports were available, supports were lacking to facilitate physician engagement and collaboration with clinical care teams in the implementation of standardised episode of care pathways. The extent to which clinical leaders and hospital administrators were able to engage physicians to collaboratively implement pathways varied by QBP and by hospital.

“*My guess is most clinicians have never read a QBP clinical handbook. So, they can’t make change if people don’t read them.*”

Source: Senior Executive, QBP Adoption Supporter, Level 2 – 018

“P: *I’m guessing I wouldn’t have to walk very far down the hall before I could find a doctor who would be grumpy about this or that in terms of what is in there. But I don’t think that is the big problem. The big problem is, what mechanisms and what tools do we have in place to relentlessly drive towards compliance standardisation? And it’s very difficult because these guys, they don’t work for me.* R: *The physicians?* P: *Yes.* R: *(Laughs.) Right, I suppose.* P: *It’s the truth. They don’t.* R: *They are not employees, in that sense, you mean?* P: *Not only are they not employees, they are essentially tenured. So, not only is it very difficult to give them direction, it’s very difficult to do anything when they don’t comply with it*.”

Source: Senior Executive, Hospital, Level 3 – 024

“*And so, that is our challenge, and we want to do better. We want to do well. We’re struggling. We keep going back and we meet with one group of docs, another group of docs, our Emergency docs. And to date, we have not found the magic to trigger it because it seems so complex, like I said, with other systems involved. So, we’re going to continue trying because we want to do well, but it is very difficult*.”

Source: Senior Executive, Hospital, Level 3 – 042

“*I think what I originally was hoping for, looking at the QBPs, I think there was hope to have a little stronger clinical engagement and how do you engage clinicians better and use existing infrastructures in the province to help create communities of practice. I don’t think that really materialised. And I think that is what we’re seeing now. It’s great that you throw a best practice at me. It’s great that you show me the baseline results. It’s great that you pay me an average price. But I have no idea how to move this forward.”*

Source: QBP Designer, Ministry of Health, Level 1 – 005

### MAIN FINDING 2: There was a mismatch between the provincial adoption supports developed in response to the policy (handbooks with evidence-based episode of care pathways) and those needed by different hospital leadership groups

Insufficient stakeholder input in planning for the implementation of QBPs led to development of adoption supports that were not effective for all involved. Stakeholders perceived incomplete, inadequate and/or inauthentic consultation and collaboration between the key groups involved (e.g. policy designers, adoption supporters, hospital administrators, local health system leaders, advisory groups, clinician and patient organisations) about how best to implement QBPs. This impeded design of effective adoption supports to enable implementation and change management, which was exacerbated by a lack of funding for change management initiatives affecting the work of both adoption support agencies and individual hospitals.

“*If you pull back the camera lens, health system funding reform, including quality-based procedures, was entirely designed and primarily implemented by the Ministry in a command-and-control function with virtually no genuine engagement and collaboration with the very sector that is supposed to implement the change. There was a very wide range of meetings and groups that came together under Ministry sponsorship from time to time, but they were inauthentic and simply masquerading as mechanisms of, again, consultation, collection of evidence and guidance, and refinement of tactic and strategy. It’s in the last year the Ministry has come to genuinely appreciate that the trajectory of HSFR* [Health System Funding Reform] *in general is no longer viable, and that a fundamental recalibration is needed. And, so they’ve abandoned the old committee structure, which was representational only, it had no other real authentic function, and we’ve reconvened a new governance collaborative.*”

Source: Senior Executive, QBP Adoption Supporter, Level 2 – 020

“*You can’t just write a handbook, throw the brick over the fence and assume your job is done. And, there are pockets of change management support for different kinds of QBPs in the province. The OHA* [Ontario Hospital Association]*, for instance, partnered with the OMA* [Ontario Medical Association] *and did regional programming for clinicians on one type of QBP last year. But, the scale of change management supports that are required on a sustained and system basis are enormous. The Ministry deserves full criticism of this. They paid no attention to the change management requirements of QBP implementation*.”

Source: Senior Executive, QBP Adoption Supporter, Level 2 – 020

“*But to some degree there has not been change management dollars behind change. And fundamentally at the end of the day, I think most people understand that change actually costs to implement and then you get the benefits down the line*.”

Source: Senior Executive, Hospital, Level 3 – 023

#### Sub-finding 2a: Hospital finance administrators lacked adoption supports aimed at informing financial operations, which undermined their role in change management and impeded QBP implementation

Adoption support handbooks focused on clinical care rather than on financial operations. However, hospital finance administrators, typically responsible for the pressing task of ensuring that costs for QBPs did not exceed the set prices, required, but lacked, analytical and decision support to inform financial operations. Without these supports, and given the time pressure to implement QBPs, hospital leadership struggled to prioritise whether (and which) changes were required for each of the QBPs. Hospital administrators responsible for finances felt there was no systematic approach to support them in their role in the change management process, or to facilitate interaction between the clinical and financial sides of hospitals.

“*I think the hospitals are pushing back and saying, slow down, because this is tougher to manage than we thought and it’s got all kinds of complication in the implementation. And even if they are done very, very well, the capacity of hospitals to really get their arms around what they should be doing with them is a process. There is quite variable capacity in hospitals to deal with that, I think. So, I think hospitals are pushing back and saying, slow down. I think the execution needs to be improved for the whole QBP and health system funding reform process.*”

Source: Senior Executive, QBP Adoption Supporter, Level 2 – 013

“*The pushback we’re getting is largely from administrators, right? This is too much, too soon, too fast. I think I’m hearing it largely from the administrative side of the house where they’re seeing the risks of not being able to predict the volumes, not being able to plan, having to change processes, all this kind of thing*.”

Source: QBP Designer, Ministry of Health, Level 1 – 006

“*Although nobody would ever say it this way, I think what it really means is, if you want us to figure out how to use these new funding formulas to maximise our revenue, we need time. And they’re really complicated and these incentives sometimes act in confusing and conflicting ways, particularly because we didn’t shift to just the straight up rate by volume funding mechanism. If we had, it would have been very clear, you’ve got to identify your admissions, or your costs are greater than your revenues, and work to reduce costs on those, and maybe reduce volumes there*.”

Source: Senior Executive, Adoption Supporter, Level 2 – 018

“*No one has shown me any information that would say, you know what, we’ve mapped out and costed the perfect care pathway for the CMG* [case mix group]*, and when you do that, this is what you should have. You should have a length of stay at this. You should have nursing coverage at that. You should have this and that. In a perfect world, this would be a cost structure. It’s still based on average performance across the province, so it’s a bit of an anomaly to think that we’ve actually tried to provide all the appropriate operational guidance, clinical guidance, to hit the mark that was, I think, fully intended at the outset*.”

Source: Senior Executive, Hospital, Level 3 – 023

#### Sub-finding 2b: There was no process to mobilise and support physician leaders and promote them as champions and change agents who could enable system wide readiness for change and adoption of QBPs

The mismatch in adoption supports was evident not only at the level of hospital financial operations, but also in the area of clinician leadership within and between hospital systems. There were no formal supports to put clinical leaders in place as champions and change agents. Although the QBP handbooks specified ideal episode of care pathways, and although the process of developing these pathways and handbooks brought together clinical leaders from around the province to participate in the technical expert advisory panels, there was no mechanism that leveraged the credibility and expertise of those leaders to encourage implementation of the care pathways in their own hospitals and across the whole hospital system.

“*Yes, the handbooks are useful, but what you really need is having strong clinical champions, having a network, having timely data on quality performance that you can provide to hospitals*.” Source: QBP Designer, Adoption Support Agency, Level 1 – 014

“*I think they vary by QBP to the extent that there are different levels of clinical engagement and support in these different service areas in Ontario. And in some areas like cancer we have a really mature and robust clinical champion and a formalised network of clinical leads that are able to do knowledge transfer really effectively through Cancer Care Ontario. That’s an outlier. And then we have less formalised clinical leadership networks, not only paid physicians, clinical leads, and then in some areas we really don’t have any infrastructure like that at all in the province. To that extent it varies because of the way that we’ve supported clinicians in being champions and engaging with their peers in knowledge transfer and exchange. And that’s just really varied by clinical area in the province. I guess I would say again I don’t know that it’s about personalities as much as to what degree we’ve supported that clinical peer knowledge translation function in the province*.”

Source: QBP Designer, Ministry of Health, Level 1 – 006

“R: *How long does it take, or did it take, to implement a new QBP from when it’s announced?* P: *Oh. Some of them were really...* R: *That was a big groan.* P: *Yeah, some of them were lickety-split easy and some of them hard, hard, hard, hard, hard.* R: *Can you differentiate between the easy and the hard and why some were easier and some were hard?* P: *Uh, yeah, yeah, yeah, I think it generally had to do with how much clinical engagement and clinical consensus you could generate and where you had the strongest physician champions. The physician champions were critical because… I mean, at the end of the day, we would have the support of the whole team, absolutely, but they don’t admit, discharge, or order*.”

Source: Senior Executive, Hospital, Level 3 – 043

#### Sub-finding 2c: Unique adoption supports intended for particular hospital leadership groups were implemented too late or not at all

Additional adoption supports aimed at hospital administrators or clinicians, such as data analytics tools and standardised clinical order sets, were envisioned and well-intentioned, but not fully implemented in time to be useful to hospitals. Hospitals in which additional supports were pursued, but which lacked stakeholder buy-in, experienced implementation failure.

“*In terms of the systems of data analytics that are required to really do it well, and that hospitals and healthcare are lacking…we don’t have some of the key enablers that will be necessary to really get us to perfection or a lot closer to perfection than we are today*.”

Source: Senior Executive, Hospital, Level 3 – 024

“*They would rather hand-write all the orders, and for the life of me, I can’t figure out why. They would rather scrawl and write exactly what they want, rather than go through this lovely pre-printed thing, check, check, yes, no, this is how it should be, and I don’t understand it. We have put them out over and over again.* R: *The pathway?* P: *We have launched... the pathways, yes* R: *The order sets?* P: *The order sets. We have launched them. We have re-launched them. We have gone around and we have talked to our internal medicine specialists, our hospitalists, our cardiologists and everybody and say, they are right here in Emerg. Oh, you know, when I go there and open the door, they are never there. And we go back to Emerg and we put them in red, and then we put up big signs, flashing light bulbs, like the blue light special. We do everything we can, and they won’t get used*.”

Source: Senior Executive, Hospital, Level 3 – 042

“*You’re in the process of already creating standardised order sets for those. So, much of what drives the care that the patient gets is based on what the physician orders and so these order sets are the tool that really enhances the success of the QBP*.”

Source: Senior Executive, Hospital, Level 3 – 030

#### Sub-finding 2d: Monitoring and evaluation tools to assess the uptake of QBPs, and their impact, were not part of the universally available adoption supports

Although there has been some evaluation of the extent to which QBPs are implemented, it has been neither systematic nor comprehensive. Instead, it focused on hospital self-reports of QBP implementation. Indicators are lacking to enable comprehensive and ongoing evaluation of (1) the scale of QBP adoption, (2) the degree to which clinicians adhered to episode of care pathways, or (3) the effect of such adherence on patient outcomes.

“*There is virtually no provincial understanding about spread and scale of QBP adoption but for some heat maps that have been sponsored by Local Health Integration Networks. That’s the only mechanism that allows us to evaluate the scale of QBP adoption in the province*.”

Source: Senior Executive, Adoption Supporter, Level 2 – 020

“*I don’t know the measures that are out there to even tell me that some of them have been implemented, other than a process measure that says someone wrote a handbook. Nobody is measuring the quality of delivery according to best practice, so how would we know?*”

Source: Senior Executive, Adoption Supporter, Level 2 – 021

“*There are some indicators, yes, absolutely, but there are many indicators that don’t exist. And, then this gets to a more kind of systemic issue, which is there’s a sense that the sector is overwhelmed in that it’s asked to conduct so much performance measurement, and all of it is given equal importance, that the sector simply doesn’t know which priority the government feels is more significant over the other. Some indicators are publicly reported, others are for boards only. Others are for management teams only. But, it’s not just QBP indicators, but there are hundreds and hundreds of indicators for completely different quality improvement, quality assurance initiatives that compete with QBP, and so you have a confounding situation on your hands*.” Source: Senior Executive, Adoption Supporter, Level 2 – 020

“*I’m not sure that anybody really has told us what the desired effect is. I don’t mean to be flippant but I just think it’s assumed all these wonderful things will happen. I think it’s early days to tell, to tell you the truth. I’m not sure they’re going to be measured locally. I think it’s almost at the system level. I would say to you at the gross system level for a primary unilateral hip or unilateral knee, are your costs stable, has your length of stay come down, and what are your outcomes?* R: *Do you collect those data, the ones you just mentioned?* P: *There’s very little outcome data available to us for anything that we do with patients. That's one of the things I think the whole system grapples with. We have lots of process indicators. We don’t have a lot of outcome indicators*.”

Source: Senior Executive, Hospital, Level 3 – 022

### MAIN FINDING 3: In response to QBPs, hospitals sometimes focused more on containing the costs of care than on improving adherence to each of the evidence-based clinical recommendations in the episode of care pathways, potentially undermining the policy intent

When QBPs were implemented, their stated goal was to address unwarranted variation. They were both a funding reform aimed at standardising costs and a quality of care initiative aimed at standardising clinical processes. Many hospitals struggled to simultaneously address these twin goals, perceiving them as conflicting or creating tension, rather than as complementary or aligned. Consequently, some hospitals focused more on one aspect of the reform (standardising cost) than on the other (standardising care). The immediate route to addressing cost-pressures of QBPs was not often perceived as flowing through the complete implementation of care pathways. Rather, quick fixes to address shortfalls instead included reducing length of stay when possible or shifting funds from other streams.

“*People are saying oh, it’s just too complex, too complicated, I’ll leave it to the finance people to deal with. And, so, I think you kind of lose that emphasis on quality once you begin to disengage, I think, for people that are in that quality side of the house.*”

Senior Executive, Hospital, Level 1 – 008

“*We use this network of physicians, and clinical leadership, and stuff like that, and we use them helping us guide the strategy of all this, but the ministry uses the CFOs* [Chief Financial Officers] *to help guide all the strategy. They’re more interested in, make it simple, make sure my hospital doesn’t get hosed somehow, and I get way more money than I used to have*.”

Source: Senior Executive, Adoption Supporter, Level 2 – 017

“*… one of those key things this has done has been a catalyst for conversations between the clinical and administrative sides of the hospital. But having said that, they’re not necessarily seeing eye to-eye, right? (Laughter) So, I think it surfaces as some tensions as well*.”

Source: QBP Designer, Ministry of Health, Level 1 – 006

“*So, we got a reduction in volume of cases, but the math would say that they were less complex cases so they should cost you less and we’re going to give you less funding. But the reality is a hip patient still went into a bed, there was still a nurse, there was still a meal delivered, there were still all these things. The length of stay didn’t drop the bottom out. So, it was a very mathematical thing to suggest that it cost us less to do the same number of hips the next year that we did last year, and yet funding went down, right?*”

Source: Senior Executive, Hospital, Level 3 – 023

“*There’s lots of change, for sure, right? And, layoffs and closure of… winding down of, FTE* [full-term employment] *positions is absolutely one of them, and so is hiring new positions and new people. So, it’s never fair to think that… it might be a cut in and of itself in that moment in time, but at an organisational basis and on a regional basis and on a provincial basis, there’s still continued growth overall. So, there’s change happening, but to the employees who it affects, yeah, it’s pretty darned important*.”

Source: Senior Executive, Adoption Supporter, Level 2 – 020

“*Typically what happens is there’s a QBP handbook comes out and the folks start to take a run at it, and they go, ‘our length of stay is a little high’ and things like that, and they do go to work on that and they bring it in line*.”

Source: Senior Executive, Hospital, Level 3 – 023

“*I think there’s probably some indicators we’re getting better outcomes. My only point is, nobody has that conversation. When you’re actually a hospital* [executive]*… when we get our numbers, I don’t hear everybody rushing into my office saying, oh my God, we did so well on QBPs this year, we had such better outcomes of patient experience. I hear from my colleagues, oh, how did you do on QBPs, did you lose or did you gain money. Then, it’s not a quality indicator*.”

Source: Senior Executive, Hospital, Level 3 – 031

“*If you don’t deliver the QBPs, you have to give the money back. If you deliver more than what you are funded for, you are not paid for it. So, for planned care, yes, we can cap at that. For unplanned care, we have no ability to cap.* R: *So, what do you do? Where do you get the money?* P: *Well, it has to come from the hospital. We have to balance the bottom line.* R: *So, you take it out of the globe?* P: *We take it out of the globe*.”

Source: Senior Executive, Hospital, Level 3 – 025

## Discussion

We found substantial variation in how hospitals responded to the QBP hospital funding reform. Our findings suggest that three factors affected the degree to which the QBP initiative was successfully implemented, namely complexity of the changes required, internal capacity for organisational change, and availability and appropriateness of targeted external facilitators and supports to manage change.

Regarding complexity of changes required, as highlighted in sub-findings 1a and 1b, surgical QBPs were easier to implement than medical QBPs. This was partly because medical QBPs involve a higher degree of clinical uncertainty and larger multi-disciplinary clinical teams with “*disparate groups of physicians and directors*”, as compared to surgical QBPs with their “*neatly defined*” episodes of care and smaller uni-disciplinary clinical teams.

The role of internal change capacity is illustrated in main finding 1, and sub-findings 2a, 2b, and 2c. Successful implementation was affected by whether there was sufficient internal capacity for managing change, such as local QI supports and/or informatics infrastructure (e.g. larger academic hospitals often benefitted from decision support systems, quality improvement teams, and effective leadership, whereas smaller hospitals lacked some of these components) and whether there is a receptive context [21, 22] and tension for change in each hospital (e.g. other issues were higher priority, for example, creating community capacity to reduce alternative level of care beds, rather than implementing the QBP clinical handbooks).

Targeted external supports provided by condition-specific agencies and provincial networks, such as for cancer and stroke, as described in sub-finding 1b, helped to facilitate implementation by leveraging existing relationships across the health system to identify and enable best practices, whereas other QBPs, such as pneumonia, lack networks.

As depicted in Table 1, our data suggest that, in general, greater external supports were needed when the complexity of the required changes was high and/or when the hospital’s internal capacity for change management was low. Fewer external adoption supports were required to achieve desired changes for QBPs involving more predictable care pathways, a narrower range of health professionals involved in care delivery, more internal capacity for change management, existing advocacy groups, and/or evidence to indicate why implementation should be a priority.

**Table 1:** Relationship between complexity of change, internal capacity for change, and external adoption supports

		Internal capacity for change	
		High	Low
Complexity of change	High	Less external support	Most external support
	Low	Least external support	Least external support

Implementation of quality-based procedures was affected by the interaction between complexity of change required, internal capacity for change and external adoption support available to facilitate change. In general, greater external supports were needed when the complexity of the required changes was high and/or when the hospital’s internal capacity for change management was low

One interpretation arising from the interplay between these factors is that policy-makers might consider restricting QBPs to areas where the changes required are less complex, and where there are known resources existing within hospitals, or in the broader system, to facilitate the necessary change management processes to achieve the desired patient care and outcomes. Policy-makers might also consider discontinuance [22] of those QBPs for which the intersection of these three factors is insufficient to optimise the chances of successful implementation. Other options for policy-makers might include grading the intensity of the adoption supports required to match the complexity of the QBP care pathway, or bringing external supports into organisations with weak internal capacity to increase their ability to deal with QBPs at all.

The challenges of standardising care within different clinical groups that involve fundamentally different microsystems do not seem to have been adequately considered in the design of the QBP adoption supports. Policy designers and adoption supporters appeared to over-estimate the capacity of some hospitals to manage change, including the receptive context for change, resulting in incomplete implementation efforts in a number of hospitals. Many hospitals were under-resourced to manage this type of change, especially without involvement of relevant external agencies with established capacity to engage clinicians and provide useful supports. It is possible to conceive of a situation where healthcare systems already have the full range of analytic decision support and change management enablers in place. However, it is often challenging to justify extensive investment in supports without the motivation of a major policy reform like QBPs. Given this dynamic, system-wide interventions are likely to limp along, unless there is substantial investment up front in the change management infrastructure to support implementation and to assess its effect on outcomes of interest. However, this means that policy designers and implementers will need to make the case for these supports, which will inevitably reduce the attractiveness of the policy change itself as its overall costs increase.

Our research found no qualitative evidence that the episode of care pathways described in QBP handbooks are being consistently used across hospitals. This would be expected given that there was neither an expectation that hospitals report the degree to which they are using the pathways nor a mechanism by which to do so. Since patient outcomes were not directly connected to payment [4], QBPs in which revenues were thought to exceed expenses did not usually draw the attention of hospital leadership, irrespective of whether the patients received care in a manner concordant with the recommendations in the handbooks. In contrast, QBPs in which expenses were estimated to exceed revenues were prioritised for action. In the absence of internal hospital capacity or external adoption supports for financial decision support (i.e. costing the care pathway and identifying outliers), those responsible for implementation of QBPs in hospitals tended to focus on easily at hand blunt tools and datasets, such as total cost per episode or length of stay. When length of stay could not be addressed for lack of adequate financial decision support resources, hospital leadership took measures to balance the budget through other mechanisms (e.g. closing beds or shifting funds from other funding streams). In general, ‘successful implementation’ of QBPs became shorthand for making just enough changes to ensure expenses for relevant patients were not greater than revenues, as opposed to identifying ways to improve patient outcomes by adhering to the episode of care pathway recommended in the handbooks.

Another possible interpretation for our results arises from the effect of policy drift on implementation. As described elsewhere [4], prior to implementation, the intended hospital funding mechanism changed substantially over time. This occurred through a lack of fidelity to the originally conceived programme theory and subsequent policy drift, leading to a cascade of changes that affected implementation of QBPs. New policies, such as hospital funding reform innovations, often roll out according to a ‘rule of thumb’ that roughly follows a sequence of discrete stages in the policy cycle [23]. These stages include (1) problem identification, (2) agenda setting, (3) policy design/formulation, (4) policy adoption, (5) policy implementation, and (6) policy monitoring and evaluation. The policy cycle is not linear, and for a policy to be successful, policy implementation must be thought of simultaneously with policy formulation. As with policy drift, misalignment at any point in the policy cycle or failure at the policy design stage to accurately predict how a policy will be implemented, can lead to conflicted success, precarious success or policy failure [24]. This research demonstrates the problems that can arise when there is a mismatch in the policy cycle between the policy formulation and the adoption supports provided to enable policy implementation. However, when policy goals drift too, as occurred with QBPs [4], overall effectiveness of the policy will not necessarily be a function of the adoption supports alone. Because the policy goals were unclear and the programme theory under-specified, it was difficult to identify what support hospitals needed to implement QBPs and to provide appropriate supports to enable adoption.

### Relation to prior work

We know of only two published studies specific to QBPs, one evaluating healthcare leaders’ early responses to implementing QBPs [25], and the other evaluating the dynamics of policy design and implementation [4].

A key debate in the policy implementation literature is the top-down versus bottom-up approach to policy-making [26, 27]. Our findings align with studies that recognise the limits of top-down policy-making, the need to involve and consult with stakeholders, and the risk of unintended negative consequences.

Prior research establishes the importance of the implementation process itself – including the utility of appropriate adoption supports – in achieving intended policy goals [22, 28–30]. Yet, policy designers tend to pay little attention to issues of implementation in general [31]. It is, therefore, important to “*focus attention on policy implementers who are capable of re-shaping policy during its implementation, with consequences for policy outcomes*” [32]. A systematic review of the evidence on diffusion of innovations has shown that the most serious gap in the literature is a lack of information on which processes enable and sustain (or not) implementation of particular innovations in health service delivery and organisation in particular contexts and settings, and whether these processes can be enhanced [22]. Our research contributes to addressing this knowledge gap by illustrating the extent to which the implementation process can influence the likelihood of success in complex system change involving a hospital funding reform.

### Strengths and limitations

Our work revealed subtleties and complexities about the research topic that may have been missed by more positivistic enquiries [33]. Acknowledging that qualitative research can be influenced by researcher bias, we made every effort to think reflexively and to openly discuss how our personal experiences and knowledge could affect the analysis process. We systematically acknowledged preconceived positions, perspectives, assumptions, values, beliefs and potential biases so they could be discussed and contested [34]. KP is a health policy analyst and researcher who previously led a systematic review of activity-based funding [3]; AB is a public policy researcher and was previously an Assistant Deputy Minister with the Ontario Ministry of Health and Long Term Care, where he worked with the key informants we interviewed through his involvement in health system funding reform; NI and DM are health system researchers and physicians affiliated with an Ontario hospital, though not one amongst our case study hospitals. We further mitigated the risk of bias through a variety of efforts to ensure validity, as described in the Methods. Another disadvantage of qualitative approaches is that their findings cannot be directly generalised in the same way as some quantitative approaches [35]. The key informants in this research came from a relatively small subset of affected hospitals chosen as cases. Likewise, this formative research occurred at a specific point in time of an ongoing implementation process and we focused specifically on QBPs as one aspect of ongoing hospital funding reforms occurring in the province. Nevertheless, the findings fit within, and contribute to, a wider literature on adoption supports specifically, system changes in general, and hospital funding reforms in particular.

### Implications

We highlight the importance of designing appropriate and targeted adoption supports as part of a fully fleshed out policy design to enable implementation, ensuring that supports are timely, account for internal change management capacity, and are appropriate to the complexity of the change. Our results also indicate the need to adjust supports if policies change due to drift, layering or other reasons.

Going forward, how can this work inform future funding reforms under consideration, such as bundled payments and other mechanisms, so that they not only achieve their cost objectives but also achieve their intended goals related to patient care? First, to avoid conflicted success (i.e. success and failure equally balanced [36]), precarious success (i.e. failure outweighs success [36]), or outright policy failure, alignment in the policy cycle is critical. This means ensuring the problem is precisely and correctly described, the policy goals are clearly established, the intervention matches the problem, the system is ready for change, enabling adoption supports are in place, and that there is a monitoring and evaluation plan in place to test the alignment. Goals and theories underlying reforms ought to be well understood so that both the intent and audience for adoption supports are clear.

Second, policy-makers ought to carefully assess the key success factors, identified in Table 1, that enable policy innovations to achieve their goals. Policy-makers who make erroneous assumptions about these key factors, or who miscalculate the degree of alignment in the policy cycle, do so at their peril, especially when the changes envisioned are expected to affect a whole healthcare system.

Consistent with the key success factors identified, we offer three observations about implementing change:

1. Consult with stakeholders to carefully assess adoption support needs. Before deciding what adoption supports are needed, policy designers ought to consult with stakeholders to assess their needs with regard to implementing desired changes. Attempts by policy designers, alone, to build new adoption supports may be constrained by a preference for one-size-fits-all approaches. Such strategies, even within one jurisdiction, are likely to fail. This highlights the importance of grounding what may seem to be a technically simple policy in a fine-grained understanding of the healthcare system.
2. Benefit from existing prêt-a porter adoption tools and communities of practice. From the outset, policy-makers would benefit from collaborating with external adoption support groups that have already built tools and communities of practice and, where such groups do not exist, take pains to accelerate their creation. Nimble and/or focused organisations (in our case, such as Health Quality Ontario, Cancer Care Ontario, and Cardiac Care Network of Ontario) are likely more able to engage directly with the field, so as to create more bespoke adoption supports.
3. Develop novel made-to-measure adoption supports in collaboration with people in the field. If new adoption supports are needed, they are best developed through extensive engagement with people working in the field so as to understand both the clinical problems and change management resources available within the healthcare system. Broad engagement with stakeholders is ideal, and the use of technical experts alone can be problematic. The ability to implement policy interventions is heavily grounded in history and context, which, by definition, varies across hospitals and jurisdictions. As such, new tools ought to be calibrated to the specific problems and to the unique needs of each microsystem.

## Conclusion

Our findings suggest that the QBP policy appears to have been developed at a high level, with the consequence that attention to implementation supports may not have been grounded in a clearly articulated and consistent programme theory. This led to a profound mismatch between the adoption supports that were likely needed, and those that were developed. Supports were focused on tools (i.e. handbooks and a toolkit) aimed at one group (i.e. healthcare professionals), but responsibility in hospitals was shared with another group (i.e. financial managers) for whom there were no customised external supports. Hospitals generally lacked capacity to standardise both costs and clinical aspects of the episode of care pathways on their own, which may have affected achievement of some policy goals. Hospital managers might have been expected to respond in a more effective and reliable manner if the policy designers and funder had made the programme theory more clear and consistent. Even still, some hospitals with less internal capacity to manage change could have benefitted from tailored external supports to ensure the policy would achieve its aims.

While the urge of policy-makers to use a financial lever to incentivise action is understandable, it is important to always be cognisant of the attendant risks, especially when the changes that are easiest to implement to earn those incentives are not the changes desired. The purpose of adoption supports developed alongside a hospital funding reform is to facilitate implementation of best-practice episode of care pathways that result in the best possible patient outcomes, rather than other changes that are merely quick fixes to address short-term budgetary concerns. If internal capacity of hospital and/or external adoption supports cannot be leveraged to meet this goal for certain QBPs, policy-makers should reconsider the approach to funding for those types of care-episodes or conditions. Additionally, if policy-makers are considering bundled payments, or other funding reforms, it is important to specifically define the clinical goals of such initiatives, reflect on whether monetary incentives are needed to encourage the necessary changes to achieve those goals, and identify the additional supports needed.

## Additional file

Additional file 1: Handbooks for Quality-Based Procedures implemented between 2012 and 2017 [5]. (DOCX 16 kb)

## Abbreviations

CEO: Chief Executive Officer
QBP: quality-based procedure
QI: quality improvement
