# Supplementary material for: Standardising costs or standardising care? Qualitative evaluation of the implementation and impact of a hospital funding reform in Ontario, Canada
Source: Health Res Policy Syst. 2018 Aug 3;16:74. doi: 10.1186/s12961-018-0353-6 (PMC6090617; doi:10.1186/s12961-018-0353-6)
Supplement: Supplementary file 1 — Handbooks for Quality-Based Procedures implemented between 2012 and 2017 [5]. (DOCX 16 kb) [file 12961_2018_353_MOESM1_ESM.docx]

| **Additional file 1**  **Handbooks for QBPs implemented between 2012-2017 [5]** | |
| --- | --- |
| 2012-13 | Primary hip and knee replacement  <http://www.health.gov.on.ca/en/pro/programs/ecfa/docs/qbp_prihipknee.pdf>  Cataract (unilateral; 2016-17 non-routine and bilateral)  <http://www.health.gov.on.ca/en/pro/programs/ecfa/docs/qbp_cataract.pdf>  Chronic Kidney Disease (CKD): <http://www.health.gov.on.ca/en/pro/programs/ecfa/docs/qbp_kidney.pdf> |
| 2013-14 | Chronic Obstructive Pulmonary Disease (COPD):  <http://www.health.gov.on.ca/en/pro/programs/ecfa/docs/qbp_copd.pdf>  Congestive Heart Failure (CHF): <http://www.health.gov.on.ca/en/pro/programs/ecfa/docs/qbp_heart.pdf>  Stroke:  <http://www.health.gov.on.ca/en/pro/programs/ecfa/docs/qbp_stroke.pdf>  Non-cardiac vascular surgery: <http://www.health.gov.on.ca/en/pro/programs/ecfa/docs/qbp_elect_aortic.pdf>  Systemic chemotherapy:  <http://www.health.gov.on.ca/en/pro/programs/ecfa/docs/qbp_chemo.pdf>  Gastrointestinal Endoscopy:  <http://www.health.gov.on.ca/en/pro/programs/ecfa/docs/qbp_gi.pdf> |
| 2014-15 | Hip Fracture:  <http://www.health.gov.on.ca/en/pro/programs/ecfa/docs/qbp_hipfracture.pdf>  Pneumonia: <http://www.health.gov.on.ca/en/pro/programs/ecfa/docs/qbp_pnemonia.pdf>  Tonsillectomy: <http://www.health.gov.on.ca/en/pro/programs/ecfa/docs/qbp_tonsil.pdf>  Neonatal jaundice: <http://www.health.gov.on.ca/en/pro/programs/ecfa/docs/qbp_jaundice.pdf> |
| 2015-16 | Knee arthroscopy:  <http://www.health.gov.on.ca/en/pro/programs/ecfa/docs/qbp_knee_arthro.pdf>  Cancer surgery (prostate, colorectal) <http://www.health.gov.on.ca/en/pro/programs/ecfa/docs/qbp_cancer_surgery.pdf> |
| 2016-17 | Cancer surgery (breast, thyroid)  <http://www.health.gov.on.ca/en/pro/programs/ecfa/docs/qbp_cancer_surgery.pdf> |
